# Prevalence and disparities in sexual and reproductive health of women of reproductive age (20–49 years) in China: A national cross-sectional study

**DOI:** 10.7189/jogh.14.04149

**Published:** 2024-09-20

**Authors:** Tian Tian, Rui Yang, Yu Fu, Zehong Zhou, Weiping Qian, Jian Zhang, Ze Wu, Lei Jin, Xueqing Wu, Cuilian Zhang, Beihong Zheng, Jichun Tan, Zhiming Zhao, Shan Luo, Yuanyuan Wang, Rong Li, Liu Ping, Jie Qiao

**Affiliations:** 1 Centre for Reproductive Medicine, Department of Obstetrics and Gynaecology, Peking University Third Hospital, Beijing, China; 2 National Clinical Research Centre for Obstetrical and Gynaecological Diseases, Beijing, China; 3 State Key Laboratory of Female Fertility Promotion, Beijing, China; 4 Key Laboratory of Assisted Reproduction (Peking University), Ministry of Education, Beijing, China; 5 Beijing Key Laboratory of Reproductive Endocrinology and Assisted Reproductive Technology, Beijing, China; 6 Guangzhou Institute of Paediatrics, Guangzhou Women and Children’s Medical Centre, Guangzhou Medical University, Guangzhou, China; 7 Department of Obstetrics and Gynaecology, Guangzhou Women and Children’s Medical Centre, Guangzhou Medical University, Guangzhou, China; 8 Department of Reproductive Medicine, Peking University Shenzhen Hospital, Shenzhen, Guangdong, China; 9 Department of Obstetrics and Gynaecology, International Peace Maternity and Child Health Hospital, School of Medicine, Shanghai Jiaotong University, Shanghai, China; 10 Department of Reproductive Medicine, The First People's Hospital of Yunnan Province, Kunming, China; 11 The Affiliated Hospital of Kunming University of Science and Technology, Kunming, Yunnan, China; 12 NHC Key Laboratory of Periconception Health Birth in Western China, Kunming, Yunnan, China; 13 Reproductive Medicine Centre, Tongji Hospital, Tongji Medical College, Huazhong University of Science and Technology, Wuhan, Hubei, China; 14 Reproductive Medicine Centre, Children's Hospital of Shanxi and Women Health Centre of Shanxi, Affiliate Hospital of Shanxi Medical University, Taiyuan, Shanxi, China; 15 Reproductive Medical Centre, Henan Provincial People's Hospital, Zhengzhou, Henan, China; 16 Reproductive Medicine Centre, Fujian Provincial Maternity and Children's Hospital, Affiliated Hospital of Fujian Medical University, Fuzhou, Fujian, China; 17 Center of Reproductive Medicine, Department of Obstetrics and Gynaecology, Shengjing Hospital of China Medical University, Shenyang 110022, Liaoning, China.; 18 Department of Reproductive Medicine, The Second Hospital of Hebei Medical University, Shijiazhuang, Hebei, China; 19 Division of Reproductive Medical Centre, West China Second University Hospital of Sichuan University, Chengdu, Sichuan, China

## Abstract

**Background:**

Ensuring women’s sexual and reproductive health (SRH) is a fundamental human right and key to 2030 agenda of the UN Sustainable Development Goals (SDGs), yet limited evidence exists on SRH in China, including national estimates and disparities of women’s SRH experiences, gynaecological diseases, and sexually transmitted diseases (STDs).

**Methods:**

A national cross-sectional survey based on a multistage stratified sampling from 15 provinces of China was performed from May 2019 to April 2021. A total of 12 815 reproductive-aged (20–49 years) women were involved. The SRH experiences (including age at menarche, age at first sexual activity, history of abortion, miscarriage, recurrent miscarriage, stillbirth, age at first delivery, types of delivery), the history of gynaecological diseases and STDs, as well as the environmental factors of participants were investigated. Human development index (HDI) was utilised to categorise and describe the socioeconomic status of the regions. The prevalence rates of diseases were compared among different HDI regions.

**Results:**

We observed a decrease in the mean age at menarche, an increase in the proportion of women who became sexually active before 20, and a modest rise in mean age at first childbirth across generations. Age-standardised prevalence estimates of miscarriage, recurrent miscarriage, artificial abortion, ectopic pregnancy, and stillbirth were 9.3, 1.4, 55.7, 3.3, and 2.1%, respectively. Approximately 50% of participants reported a history of gynaecological diseases, with vulvovaginitis, cervicitis, and pelvic infection diseases being the most prevalent. The overall prevalence of STDs was estimated at 22.2‰, with mycoplasma genitalium infection having the highest reported prevalence. Disease prevalence varies across HDI regions.

**Conclusions:**

Women's SRH behaviours and experiences have evolved, along with shifts in the spectrums of gynaecological diseases and STDs in China. Urgent recalibration of health care policies and disease control strategies is necessary, aligning them with women's changing SRH needs, ultimately ensuring their reproductive health and rights.

Sexual and reproductive health (SRH) is an indispensable step towards achieving the UN Sustainable Development Goal (SDG) target of ensuring universal access to sexual and reproductive health care services by 2030 (SDG 3.7) [1]. While the global health community has made tremendous efforts to promote women’s SRH by improving services for family planning, maternal and newborn care, and prevention and treatment of HIV/AIDS, it is time to look beyond these standard aspects of SRH and recognise facets that have been left unaddressed [2].

Representing over a fifth of the global population, China has made concerted efforts in achieving SRH-related SDGs over the past few decades, which is evident in the dramatic reductions in maternal and child mortality and HIV infection [3]. With the global shift in SRH politics and the emerging challenges of decreased and delayed fertility intention, both within China and globally [3,4], it is important to have a comprehensive understanding of how the key milestones in women’s reproductive life, such as the age at menarche, the initiation of sexual activity, and the timing of first childbirth, have evolved. Understanding these temporal changes is instrumental for informing policy and tailoring programmes that align with women’s current SRH needs.

However, there is a noticeable data gap on SRH experiences and conditions. For example, population-based estimates of miscarriage, recurrent miscarriage, ectopic pregnancy, and stillbirth rates are limited in China. These experiences could exert devastating repercussions extending beyond the women themselves also to affect their families. Existing studies conducted in China may provide some insights, but they primarily focused on hospital-based data, which might hinder the strength of the findings [5–7]. Updated data on gynaecological diseases also remains inadequate. Unlike diseases such as gynaecologic cancer, endometriosis, or polycystic ovary syndrome (PCOS), which have garnered most of the spotlight [8–10], many disorders within the spectrum of gynaecological diseases like vulvovaginitis and cervicitis are often overlooked. The situation is similar for STDs. While the data concerning STDs, especially HIV/AIDS, have been well-documented at both global and regional levels, numerous STDs other than HIV/AIDS still lack attention in terms of public awareness and research. Therefore, there is an urgent need for nationally representative data on these neglected areas of SRH to achieve SDGs.

Equity in the allocation of medical resources plays a crucial role in human society's healthy and sustainable development. Though China has made significant progress in its medical and health care system, disparities persist in the provision of medical services, notably between urban and rural areas and across economic regions [11], posing structural barriers to the intrinsic equity component of SDG 3 that calls for universal access to sexual and reproductive health care. In line with this commitment, studies that could uncover gaps, disparities, and areas falling short in serving women's SRH needs are warranted to advance SRH health care provision.

In the absence of population-level evidence on women’s SRH, we conducted a national cross-sectional survey involving 12 815 reproductive-aged women from 15 provinces of China. This study aimed to present national estimates and disparities of women’s SRH experiences, gynaecological diseases, and STDs to identify higher-risk populations and factors that could increase their vulnerability and thereby help inform China and other countries with similar contexts of priority setting at the national or regional level and to stay on track with SRH-related targets in the SDGs era.

## METHODS

### Study design and participants

This study used data from the 2020 China Fertility Survey of Married Women (CFSMW) programme. The 2020 CFSMW was a nationwide cross-sectional survey conducted in China. A multistage stratified sampling was performed from May 2019 to April 2021. Fifteen provinces were initially selected. Subsequently, three districts from each province were selected according to their degree of urbanisation and population size. Two to four villages/residential areas from each district were further selected by a random sampling method. Finally, 100 eligible women aged 20 to 49 years who had lived in the selected area for more than six months were invited to participate in the survey. The analysis included a total of 12 815 reproductive-aged women who completed the survey. A detailed protocol for the 2020 CFSMW was described in our previous publication [12].

### Data collection

For each participant, a face-to-face structured survey was conducted by well-trained investigators to collect detailed information covering different aspects of participants’ reproductive health. The survey included basic demographic information such as birthdate, ethnic groups, educational levels, family income, medical insurance types, etc. Additionally, participants’ SRH experiences were recorded, including age at menarche, age at first sexual activity, history of abortion, miscarriage (spontaneous loss of a clinical pregnancy before 22 completed weeks of gestational age), recurrent miscarriage (≥2 miscarriages), stillbirth, age at first delivery, types of delivery, etc.

The history of diseases included gynaecological diseases and STDs. Gynaecological diseases included vulvovaginitis, cervicitis, pelvic inflammatory diseases (PID), genital tuberculosis, uterine myoma, endometriosis, genital malformation, and diminished ovarian reserve (all conditions should be diagnosed by a clinician). STDs included HIV, gonorrhoea, syphilis, condyloma acuminatum, genital herpes, mycoplasma genitalium infection, *Chlamydia trachomatis* genital infection, toxoplasma gondii and cytomegalovirus.

The environmental factors included harmful behaviours (i.e. active/passive smoking, drinking), harmful environmental exposure (i.e. acoustic pollution, pesticides, toxic metals, and chemicals), and occupational factors (i.e. work stress and intensity).

### Classification of socioeconomic status and indicators

Human development index (HDI) was utilised to categorise and describe the socioeconomic status of the regions in this study. Human development index is a composite index focusing on human development: the ability to lead a long and healthy life, the ability to acquire knowledge, and the ability to achieve a decent standard of living, which is a critical indicator required by the SDGs for assessing inequality [13]. The 15 provinces were divided into six HDI regions based on the percentile of the HDI: very-low HDI regions (≤10th percentile), low HDI regions (>10th to 25th percentile), lower-middle HDI regions (>25th to 50th percentile), upper-middle HDI regions (>50th to 75th percentile), high HDI regions (>75th to 90th percentile), and very-high HDI regions (>90th percentile)[14] (Table S1 in the **Online Supplementary Document**).

### Statistical analysis

We estimated crude and weighted prevalence of various SRH experiences, gynaecological diseases, and STDs. The crude prevalence was calculated as the ratio between the number of diagnosed participants and the number of all participants in this survey. Standard errors (SEs) and 95% confidence interval (CI) for the prevalence were determined by using the Taylor series linearisation method [15]. The weighted prevalence was calculated using sampling and post-stratification weights [12]. Sampling weights at each stratum were equal to the reciprocal of the corresponding sampling probability. The post-stratification weights took into account two dimensions: the six HDI groups and six age subgroups. The weighted prevalence was calculated based on the 36 strata groups using data from the seventh national population census of 2020 in China as the standardised population [16] (Table S2 in the **Online Supplementary Document**).

For continuous variables, such as age at menarche and age at first delivery, the normality was tested by Kolmogorov-Smirnov test. Mean ± SD was used to describe the levels when the variable was normally distributed. When the variable did not conform to a normal distribution, median and interquartile range (P25 – P75) were used. The distributions of categorical variables, such as smoking, drinking, and other environmental factors, were compared between HDI groups by using the χ^2^ test. To estimate the trend of prevalence across female age and HDI, a Cochran–Armitage χ^2^ test was conducted for trend analyses.

All analyses were performed using R software (version 4.1.0, Austria, URL https://www.R-project.org/). The tests were considered statistically significant when a two-sided *P*-value was less than 0.05.

In addition, the sample size was calculated by PASS 13.0 based on previously reported prevalence and their 95% CIs of some major gynaecological diseases and STDs. For example, a sample size of 8149 produces a two-sided 95% CI with a width equal to 0.004 when the prevalence of syphilis is 0.8% [17,18] and a sample size of 5077 produces a two-sided 95% CI with a width equal to 2% when the prevalence of miscarriage is 15% [19]. Therefore, the sample size of our study 12 518 could satisfy the study design.

## RESULTS

**Figure 1:**
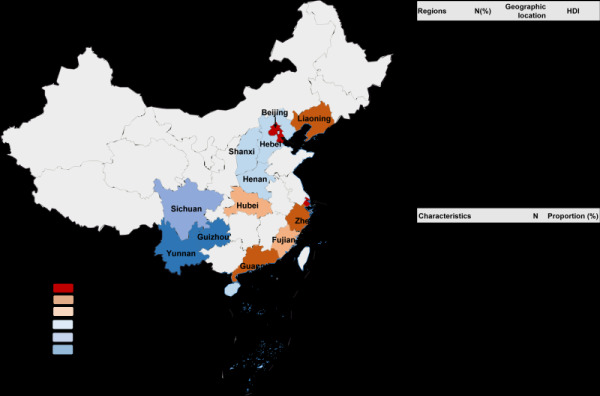
Regions and key characteristics of participants included in the study.

As shown in **Figure 1**, 12 815 participants aged from 20 to 49 years from 15 provinces of China were included in the analysis. The mean age of all participants was 36.8 ± 7.1 years. Among the participants, 518 (4.0%) were aged 20 to 24, and 2222 (17.3%) were aged 45 to 49. A total of 6932 (54.1%) women were urban citizens, and 5883 (45.9%) were rural citizens. The average body mass index (BMI) of the participants was 23.2 ± 4.1 kg/m^2^. The majority of women (93.8%) were Han ethnicity. Over half of the participants (55.5%) had attained a college education or higher. Based on the HDI groups, 26.2% of the participants were from very-high HDI regions, while 11.0% were from very-low HDI regions. Detailed characteristics of the women are presented in Table S3 in the **Online Supplementary Document****.**

**Figure 2:**
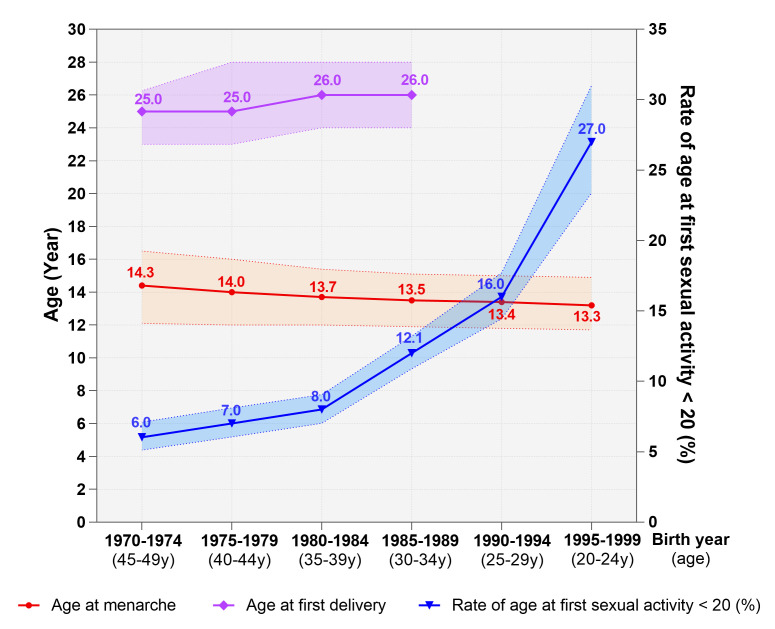
Generational trends of sexual and reproductive health milestones in China.

**Figure 2** depicts the temporal changes in participants’ reproductive milestones over time. The mean age at menarche displayed a downward trajectory, decreasing from 14.3 years for women born in 1970–1974 to 13.3 years for those born in 1995–1999. Of the women born between 1970–1974, only 6.0% had become sexually active before 20 years old. This rate has been rising dramatically with time, reaching 27.0% in women born in 1995–1999. The mean age at first childbirth showed a modest upward trend across generational cohorts. The mean age for a woman’s first delivery was 25.0 for those born between 1970–1974 and 1975–1979. However, this figure rose to 26.0 for women born between 1980–1984 and 1985–1989.

Furthermore, as **Table 1** presents, 33.5% of women reported experiencing irregular menstruation. The age-standardised prevalence rate was estimated to be 9.3% for miscarriage and 1.4% for recurrent miscarriage. With the increase in HDI, the recurrent miscarriage rate displayed a decline, diminishing from 2.0% in the very-low HDI regions to 0.8% in the very-high HDI regions. As for other SRH experiences, the age-standardised prevalence rates of artificial abortion, ectopic pregnancy, and stillbirth were estimated to be 55.7%, 3.3%, and 2.1%, respectively. Significant patterns were observed across different HDI regions, revealing declining trends in the prevalence of ectopic pregnancy and stillbirth (*P*_trend_<0.001). Specifically, regions with very-low HDI exhibited considerably higher rates of ectopic pregnancy at 5.9% compared to a rate of 2.1% in areas with very-high HDI. Similarly, the prevalence of stillbirth was 3.1% in very-low HDI regions compared to a rate of 1.2% in very-high HDI regions. Also, we reported a standardised Caesarean delivery rate of 33.5% (95% CI = 31.8–35.5%) in women of reproductive age. It is worth noting that Caesarean delivery rates were higher in very-low and very-high HDI regions when compared to other HDI regions.

**Table 1:** Prevalence estimates of sexual and reproductive health experiences in China

Sexual and reproductive health experiences	Level	N	Overall		Human development index							
			**Crude prevalence (%)**	**Adjusted prevalence (%)**	**Very-low**	**Low**	**Lower-middle**	**Upper-middle**	**High**	**Very-high**	***P*-value**	***P*_trend_**
Menstrual cycle*	Regular	8688	67.8 (67.0–68.6)	66.5 (64.5–68.6)	67.1	61.0	61.4	70.1	70.4	67.4	<0.001	0.004
	Irregular	4127	32.2 (31.4–33.0)	33.5 (32.0–35.1)	32.9	39.0	38.6	29.9	29.6	32.6		
Miscarriage†		927	8.3 (7.8–8.8)	9.3 (8.3–10.6)	9.4	13.3	9.8	8.4	7.5	7.6	0.015	0.042
Recurrent miscarriage†		160	1.4 (1.2–1.7)	1.4 (1.1–2.0)	2.0	1.6	1.4	1.4	0.9	0.8	0.058	<0.001
Artificial abortion†‡		6348	56.8 (55.9–57.8)	55.7 (53.5–58.2)	59.3	61.3	53.2	55.8	54.5	50.9	<0.001	0.087
Ectopic pregnancy†		359	3.2 (2.9–3.6)	3.3 (2.8–4.2)	5.9	3.6	3.1	3.4	2.9	2.1	<0.001	<0.001
Stillbirth†		225	2.0 (1.8–2.3)	2.1 (1.7–2.9)	3.1	1.5	3.3	1.8	1.7	1.2	<0.001	<0.001
Types of delivery§	Vaginal	6443	60.8 (59.8–61.7)	64.4 (61.7–67.4)	62.4	67.2	65.5	63.3	64.4	59.2	<0.001	0.005
	Caesarean	3992	37.7 (36.7–38.6)	33.5 (31.8–35.5)	35.8	31.5	30.5	34.7	34.7	39.5		

*Denominator: whole population.

†Denominator: women with a history of pregnancy.

‡Artificial abortion included drug-induced abortion and induced abortion.

§Denominator: women with delivery history.

**Figure 3:**
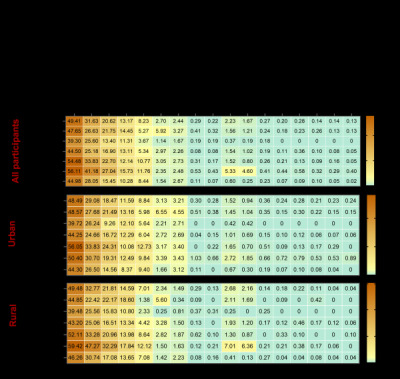
Heatmap of prevalence (% estimates for gynaecological diseases and STDs by HDI in urban and rural areas. The prevalence was age adjusted. HDI – human development index, STDs – sexually transmitted diseases.

Prevalence of gynaecological diseases was estimated. Approximately 49.4% of the study population reported a history of gynaecological diseases. Estimates shown in **Table 2** indicate that vulvovaginitis, cervicitis, and PID were the most prevalent, with adjusted prevalence of 31.6% (95% CI = 30.3–33.1%), 20.6% (95% CI = 19.6–21.8%), and 13.2% (95% CI = 12.4–14.1%), respectively. Additionally, the study assessed the overall prevalence of STDs, which was estimated to be 22.2‰ (95% CI = 19.3–26.9‰). Among the various STDs surveyed, mycoplasma genitalium infection had the highest reported prevalence, reaching a rate of 16.7‰ (95% CI = 14.3–20.8‰). **Figure 3** presents the regional disparities for gynaecological diseases and STDs across HDI regions, as well as between urban and rural participants. Notably, distributions of disease prevalence differed by HDI regions. A noteworthy pattern emerged regarding the prevalence of overall gynaecological diseases, vulvovaginitis, and cervicitis: increasing trends were observed as HDI increased from low to high. However, a twist in this pattern was observed in regions with very-high HDI, where the prevalence rates showed a decline despite the overall upward trend. More detailed information is presented in Tables S3–5 in the **Online Supplementary Document**.

**Table 2:** Prevalence estimates of gynaecological diseases and STDs in China

Gynaecological diseases and STDs	N	Crude prevalence (95% CI)	Adjusted prevalence (95% CI)
**Gynaecological diseases (%)**			
Overall	6573	51.2 (50.4–52.1)	49.4 (47.7–51.2)
Vulvovaginitis	4162	32.5 (31.7–33.3)	31.6 (30.3–33.1)
Cervicitis	2742	21.4 (20.7–22.1)	20.6 (19.6–21.8)
Pelvic infection diseases	1769	13.8 (13.2–14.4)	13.2 (12.4–14.1)
Uterine myoma	1142	8.9 (8.4–9.4)	8.2 (7.6–9.0)
Diminished ovarian reserve	365	2.8 (2.6–3.2)	2.7 (2.3–3.2)
Endometriosis	355	2.8 (2.5–3.1)	2.4 (2.1–2.9)
Genital malformation	27	0.2 (0.1–0.3)	0.3 (0.2–0.6)
Genital tuberculosis	21	0.2 (0.1–0.3)	0.2 (0.1–0.6)
**STDs (‰)**			
Overall	283	22.1 (19.6–24.8)	22.2 (19.3–26.9)
Mycoplasma genitalium infection	218	17.0 (14.8–19.4)	16.7 (14.3–20.8)
Condyloma acuminatum	29	2.3 (1.5–3.2)	2.7 (1.5–6.1)
Syphilis	19	1.5 (0.9–2.3)	2.0 (1.0–5.4)
*Chlamydia trachomatis* genital infection	17	1.3 (0.8-2.1)	2.8 (1.0–5.4)
Toxoplasma gondii and cytomegalovirus	17	1.3 (0.8–2.1)	1.4 (0.7–4.7)
Genital herpes	12	0.9 (0.5–1.6)	1.4 (0.5–4.7)
Gonorrhoea	9	0.7 (0.3–1.3)	1.3 (0.4–4.6)

CI – confidence interval, STDs – sexually transmitted diseases

We further investigated environmental factors that could affect women’s reproductive health (**Table 3**). While the rate of active smoking was relatively low among reproductive-aged women, the rate of passive smoking reached 34.9%. Higher rates of passive smoking were observed in regions with lower HDI. Regarding other harmful environmental exposures, 8.9% of the participants reported being exposed to acoustic pollution, and 3.1% of women reported being exposed to toxic chemicals. Additionally, 32.7% of women reported experiencing moderate to severe stress at work and 24.2% had high work intensity.

**Table 3:** Environmental factors of participants included in the study

Environmental factors	Level	Overall	Human development index						*P-*value	*P _trend_*
			**Very-low**	**Low**	**Lower- middle**	**Upper- middle**	**High**	**Very-high**		
**Harmful behaviours**										
Active smoking	Yes	230 (1.8)	46 (3.3)	25 (4.9)	31 (1.0)	23 (1.4)	32 (1.2)	73 (2.2)	<0.001	0.018
Active smoking frequency										
	≤1day/week	34 (0.3)	11 (0.8)	6 (1.2)	3 (0.1)	5 (0.3)	5 (0.2)	4 (0.1)	<0.001	0.944
	2–3 d/week	33 (0.3)	6 (0.4)	6 (1.2)	5 (0.2)	3 (0.2)	3 (0.1)	10 (0.3)		
	4–6 d/week	16 (0.1)	3 (0.2)	1 (0.2)	2 (0.1)	5 (0.3)	2 (0.1)	3 (0.1)		
	7 d/week	125 (1.0)	22 (1.6)	9 (1.7)	13 (0.4)	7 (0.4)	22 (0.8)	52 (1.5)		
Passive smoking	Yes	4465 (34.9)	595 (41.2)	175 (33.9)	879 (27.4)	582 (34.7)	1067 (40.0)	1169 (34.9)	<0.001	0.498
Passive smoking frequency	≤3h/d	4124 (32.2)	548 (38.9)	164 (31.8)	802 (25.1)	533 (31.8)	1011 (37.9)	1066 (31.8)	<0.001	0.514
	>4 h/d	341 (2.7)	47 (3.3)	11 (2.1)	75 (2.3)	49 (2.9)	56 (2.1)	103 (3.1)		
Drinking	Yes	1567 (12.2)	270 (19.2)	116 (22.5)	347 (10.9)	231 (13.8)	238 (8.9)	365 (10.9)	<0.001	<0.001
Drinking frequency										
	≤1day/week	1322 (10.3)	225 (16.0)	104 (20.2)	283 (8.9)	197 (11.7)	201 (7.5)	312 (9.3)	<0.001	<0.001
	2–3 d/week	143 (1.1)	21 (1.5)	6 (1.2)	28 (0.9)	23 (1.4)	25 (0.9)	40 (1.2)		
	4–6 d/week	22 (0.2)	6 (0.4)	2 (0.4)	2 (0.1)	4 (0.2)	3 (0.1)	5 (0.1)		
	7 d/week	29 (0.2)	2 (0.1)	3 (0.6)	5 (0.2)	2 (0.1)	7 (0.3)	10 (0.3)		
**Harmful environmental exposures**										
Acoustic pollution		1139 (8.9)	121 (8.6)	95 (18.4)	208 (6.5)	122 (7.3)	312 (11.7)	281 (8.4)	<0.001	0.583
Pesticides		72 (0.6)	7 (0.5)	9 (1.7)	29 (0.9)	5 (0.3)	6 (0.2)	16 (0.5)	<0.001	0.01
Toxic and harmful chemicals		396 (3.1)	34 (2.4)	98 (19.0)	63 (2.0)	44 (2.6)	55 (2.1)	102 (3.0)	<0.001	<0.001
Toxic metals		18 (0.1)	3 (0.2)	1 (0.2)	1 (0.0)	1 (0.1)	10 (0.4)	2 (0.1)	0.006	0.942
**Occupational factors**										
Stress at work	Moderate/severe	4190 (32.7)	680 (48.3)	176 (34.2)	970 (30.4)	517 (30.8)	685 (25.6)	1162 (34.6)	<0.001	<0.001
Work intensity	Moderate/severe	3107 (24.2)	358 (25.5)	133 (25.9)	735 (23.0)	319 (19.1)	764 (28.6)	798 (23.8)	<0.001	0.466

## DISCUSSION

Based on the data from a nationwide cross-sectional survey with 12 815 women of reproductive age (20–49 years), this study reveals women’s evolving SRH behaviours and experiences and presents estimates and disparities of many long-neglected SRH conditions, including miscarriage and recurrent miscarriage, ectopic pregnancy, stillbirth, gynaecological diseases, and STDs. These issues could exert a considerable impact on women’s health and well-being across their life courses, yet often remain underexplored in research and unacknowledged within the priorities of health care service provision. Our findings demonstrate the areas and the scopes of the unfinished SRH agenda and highlight the importance of ensuring universal access to SRH care.

Our study has unveiled a decreasing trend in age at menarche, which corroborates with the global consensus [20,21]. A review consisting of studies from 27 low-income and middle-income countries (LMICs) supports this downward trend within and between countries [21]. Additionally, we have noted a significant rise in the proportion of women who became sexually active before the age of 20. Research indicates that in most developed countries and some parts of Africa, over a third of unmarried young girls are sexually active [22]. According to a review, about 12% of Chinese young people aged 10–24 were sexually active, with an average age of 19.4 years at first sexual intercourse [23]. Over the past two decades, shifts in social norms and narratives related to sexuality have transformed sexual and reproductive behaviours and attitudes around the world. With the gap between the initiation of sexual intercourse and marriage growing in many countries, more individuals are now engaging in premarital sexual activities. The considerable increase in premarital sexual activity and high-risk sexual behaviours amongst adolescents may lead to a rise in unintended pregnancies [23,24]. Considering that menarche and sexual initiation are both pivotal milestones in one’s reproductive life, symbolising adulthood and reproductive potential, the downward trajectories presented in this study underscore the pressing need to provide early, age-appropriate comprehensive sex education and SRH services for adolescents to address unintended pregnancy and promote health and well-being later in life.

Our study provides the first nationwide estimates of miscarriage, recurrent miscarriage, ectopic pregnancy, and stillbirth in China using community-based data. We reported a prevalence of 9.3% for miscarriage, 1.4% for recurrent miscarriage, 3.3% for ectopic pregnancy, and 2.1% for stillbirth. Evidence on miscarriage and recurrent miscarriage vary dramatically due to heterogeneity in definitions, geographical regions, and study design. One review reported a global pooled estimate of 15.3% for miscarriage and 0.7% for recurrent miscarriage [19]. A hospital-based study reported upward trends for miscarriage and ectopic pregnancy incidence in China from 2003 to 2013 [5]. Despite the detrimental consequences of miscarriage, such as obstetric complications, subsequent infertility, poorer mental health, cardiovascular disease and venous thromboembolism [19], estimates of miscarriage rates are limited in China. Nationwide data on stillbirth also remains scarce, posing challenges in identifying vulnerable groups and providing health care services. A cross-sectional study conducted across 441 hospitals in China reported an estimated stillbirth rate of 8.8 cases per 1000 births between 2012 and 2014 [6]; another study encompassing 96 hospitals across 24 provinces reported a rate of 13.2 per 1000 births in 2015–2016 [7]. Yet, the hospital-based nature of these studies might restrict their empirical robustness. Our findings significantly contribute to bridging the evidence gap and informing targeted interventions and policies in China and countries with similar contexts.

The World Health Organization (WHO) has consistently broadened the concept of SRH to one that goes beyond mere reproductive capacity [25], thus underscoring the holistic recognition of SRH problems that could impact women’s health across their lifespans. Our findings illuminate that approximately half of women of reproductive age have experienced a history of gynaecological disease. Despite this high prevalence, many conditions within the spectrum of gynaecological diseases are often neglected in research and health care provision. For example, our study showed that vulvovaginitis emerged as a significant concern, affecting more than 30% of women. Vulvovaginitis could severely jeopardise women’s quality of life by affecting not only their physical health but their self-esteem, sexual experience, and interpersonal relationships in the long-term [26]. On another front, STDs affect women worldwide throughout their lives and can lead to various complications, including infertility and chronic pelvic pain, thus contributing to increased peripartum morbidity and mortality globally [27–29]. China has made tremendous efforts in tackling HIV, syphilis, and gonorrhoea [3]. Syphilis was identified as the most common STD in China, with morbidity increasing from 15.8834 per 100 000 in 2007 to 34.4867 per 100 000 in 2017 [30]. Previous studies focusing on specific regions or populations, such as pregnant women or female sex workers, reported cumulative prevalence rates of syphilis ranging from 0.3 to 0.8% [17,18]. Our study found a prevalence rate of syphilis at 2.0‰ among general reproductive-aged women, which was lower than other STDs, indicating a shifting STDs spectrum. Mycoplasma genitalium infection was found to have the highest prevalence, with rates of 16.7‰, followed by *Chlamydia trachomatis* genital infections and condyloma acuminatum at 2.8‰ and 2.7‰, respectively. These rates were relatively lower than previous reports in China [31–33], possibly due to the community-based rather than hospital-based nature of our study, providing a more representative epidemiology of these diseases in the general population. Therefore, there is an urgent call to action to recalibrate health care policies and modify disease control and prevention strategies based on the updated burden of diseases, aligning them with women’s evolving SRH needs, with the ultimate goal of ensuring women’s reproductive health and rights.

Previous studies have demonstrated significant disparities in the prevalence of diseases among countries with varying socioeconomic statuses [34]. This is also true in China and many other countries, where the unequal distribution of medical and public health resources remains a formidable challenge in health care management [35]. Regions with robust economic development tend to have a concentration of high-quality medical resources [3], including skilled clinicians and well-equipped tertiary hospitals, fostering superior diagnostic and treatment capabilities. Our findings revealed significant variations in the prevalence of gynaecological diseases and STDs, both across HDI regions and between urban and rural areas, suggesting that the uneven allocation of medical resources may fuel geographical and socioeconomic disparities in female reproductive health. Evidence has revealed that women and adolescents with disadvantageous socioeconomic status are less likely to utilise SRH services [36,37]. Notably, we observed an increasing prevalence of gynaecological diseases with higher HDI. This could be attributed to differential screening, as individuals in higher-HDI regions are more likely to seek health care services. Conversely, in lower-HDI areas, limited access to health care services remains a barrier to early diagnosis, resulting in lower reported prevalence rates compared to wealthier socioeconomic regions. Moreover, ruralurban migrants tend to underuse health care services, largely because their health insurance typically does not reimburse the expenses at their urban locations [38,39]. Given the dynamic changes in various factors such as social policies, population mobility, and socioeconomic structures, it is imperative to promote equitable access to women’s reproductive health care across all economic regions. Future research endeavours should focus on the intricate socioeconomic determinants of SRH, including factors such as income, education, access to health care, and other sociocultural factors. Qualitative research could be utilised to gain a deeper understanding of the experiences of underserved populations, informing the development of health care policies.

Environmental factors have been proven to play crucial roles in female reproductive health. For example, cigarette smoking and drinking could disrupt hormone levels, affect ovulation, and increase the risk of infertility, miscarriage, ectopic pregnancy, and other reproductive issues [40]. Emerging studies also demonstrated that exposure to harmful substances, such as pesticides, chemicals, and toxins, may lead to hormonal imbalances, menstrual irregularities, reduced fertility, and adverse effects on foetal development [41,42]. In this study, we found that although the active smoking rate was low, passive smoking is still a severe issue faced by Chinese women, with an estimated rate of 32.2%. The prevalence of passive smoking was reported to be 30.1% among Iranian women [43], and worldwide, 35% of female non-smokers were exposed to passive smoking [44]. Additionally, about 9% of participants reported being exposed to acoustic pollution. Our data indicated that it is essential to recognise and address these ecological determinants, such as passive smoking and acoustic pollution, to safeguard women’s reproductive health. Moreover, in our study, more than 30% of women reported experiencing moderate to severe stress at work, while 24.2% reported moderate to severe working intensity. Physically demanding or strenuous work can exert excessive strain on both the body and mind, potentially affecting reproductive health. Thus, focusing on women’s physical and mental health in the workplace should be considered an important goal in ensuring women’s SRH.

Our study presents the first nationwide estimates of many SRH experiences and conditions by utilising nationwide community-based data, which not only addresses the existing data gap but also enhances the representativeness of our findings. The implications of this study extend beyond the mere presentation of estimations. Our findings shed light on the long-neglected areas of SRH and revealed the changing spectrum of gynaecological diseases and STDs among reproductive-aged women. Moreover, our study underscores the regional disparities in SRH. Policymakers should prioritise the integration of SRH services into national primary health care (PHC) systems [45]. Additionally, efforts to promote health equity should focus on addressing the underlying social determinants of SRH, including income inequality, education gaps, and limited access to health care services, etc. Constructive strategies, such as expanding access to comprehensive sex education [46] and improving health care infrastructure in underserved areas [47] could advance SRH equity. Our study could help inform priority-setting efforts and shape evidence-based strategies and programmes in China and other countries with similar contexts to promote women’s SRH and accelerate the achievement of SDGs.

In China, the COVID-19 pandemic emerged and began spreading in January 2020. However, from 2020 to 2022, the Chinese government implemented very strict prevention policies, which helped contain the epidemic. On 11 November 2022, China relaxed its lockdown measures, leading to a rapid uptick in COVID-19 cases. Although our participants were involved from 2019 to 2021, most of the women completed the investigation before January 2020. Among 12 815 participants, only 302 participants (2.35%) were investigated after 2020. Therefore, we assume that the SRH-related rates and conditions reported in this study were not influenced by COVID-19. This raises an important concern: the results of our study likely reflect the SRH conditions before the outbreak of COVID-19. The SRH conditions after COVID-19 require further investigation.

Some other limitations of our study need to be considered when interpreting these results. First, owing to the nature of the cross-sectional study design and the questionnaire investigation, data on the history of SRH experiences, gynaecological diseases, and STDs in this study were self-reported, which may lead to recall bias and measurement bias in the estimated prevalence. Further medical record data and national surveillance data, as well as prospective study are required to validate the results of our study. Second, due to the sensitive nature of questions pertaining to SRH experiences, particularly those regarding sexual initiation, respondents’ willingness to disclose may have been affected, potentially introducing response bias. Third, considering the SRH experiences and conditions may have more significance for reproductive-aged women, especially for the women who have reproductive need, our survey focused on women aged 20 (the legal age for marriage in China) to 49, which makes prevalence reported in our study could not accurately reflect women under 20 and elder women who were older than 50. However, age-standardisation was used to mitigate some of the potential biases arising from this limitation.

## CONCLUSIONS

Through a nationwide cross-sectional survey, we presented the national estimates and disparities of SRH experience and conditions of women aged 20 to 49 years in China. National health care programmes should be tailored to meet women’s current SRH needs. Key considerations should include the evolving reproductive milestones in young women, the changing spectrum of gynaecological diseases and STDs among women of reproductive age, and maintaining SRH across the lifespan. Integration of SRH services into PHC is crucial to achieving universal health coverage (UHC) and ensuring the accessibility and affordability of health care [45]. By recognising and addressing these evolving SRH needs, we are able to promote women’s overall well-being and stay on track with SRH-related targets in the SDGs era.

## Additional material


Online Supplementary Document
