# Supplementary material for: Prevalence and disparities in sexual and reproductive health of women of reproductive age (20–49 years) in China: A national cross-sectional study
Source: J Glob Health. 2024 Sep 20;14:04149. doi: 10.7189/jogh.14.04149 (PMC11414425; doi:10.7189/jogh.14.04149)
Supplement: Online Supplementary Document [file jogh-14-04149-s001.pdf]

Title: Prevalence and disparities in sexual and reproductive health of women of reproductive age (20-49 years) in China: a national cross-sectional study

Online Supplementary Document (OSD)

Table S1 Regions of China by Human Developmental Index (HDI)

HDI	Percentile of the HDI	HDI thresholds	City/Regions
Very low	≤10th	≤ 0·667	Guizhou Qinghai Xizang Yunnan
Low	>10th to 25th	(0·667–0·708]	Anhui Gansu Guangxi Sichuan
Low middle	>25th to 50th	(0·708–0·733]	Hainan Hebei Henan Heilongjiang Jiangxi Ningxia Shanxi Xinjiang
Upper middle	>50th to 75th	(0·733–0·754]	Fujian Hubei Hunan Jilin Inner-Mongolia Shandong Shannxi Chongqing
High	>75th to 90th	(0·754–0·784]	Guangdong Jiangsu Liaoning Zhejiang
Very high	>90th	> 0·784	Beijing Shanghai Tianjin

Table S2 Post-stratification and standardized population of China

HDI Regions	Female age	Study participants	Standardized population
**Very Low HDI**	20-24	139	3060422
	25-29	307	3085817
	30-34	359	3510969
	35-39	242	2965491
	40-44	213	3228393
	45-49	147	3778298
**Low HDI**	20-24	44	5524121
	25-29	88	6743278
	30-34	101	8396849
	35-39	74	6517851
	40-44	92	6568986
	45-49	116	9248918
**Lower-middle HDI**	20-24	139	7798831
	25-29	590	9579504
	30-34	787	14046276
	35-39	668	11021543
	40-44	554	10512699
	45-49	454	12728073
**Upper-middle HDI**	20-24	34	8685809
	25-29	281	10594608
	30-34	453	16673488
	35-39	412	13005747
	40-44	321	12048838
	45-49	176	15714328
**High HDI**	20-24	80	8493448
	25-29	315	11310018
	30-34	495	14515333
	35-39	596	11877401
	40-44	579	10867683
	45-49	602	12462191
**Very high HDI**	20-24	93	1703049
	25-29	379	2371837
	30-34	818	3130467
	35-39	736	2692862
	40-44	623	2096037
	45-49	708	2101393

Table S3 Basic characteristics of participants

Characteristics	Sample Size (n)	Proportion (%)
**All**	**12,815**	**100.0**
Maternal age		
Mean (SD)	36·77 (7·13)	
Median (IQR)	36·0 (31·0-42·0)	
20-24	518	4·0
25-29	1944	15·2
30-34	3017	23·5
35-39	2731	21·3
40-44	2383	18·6
45-49	2222	17·3
Body Mass Index (kg/m^2)		
Mean (SD)	23·2 (4·1)	
<18.5	734	5·7
18.5-23.9	7528	58·7
24.0-27.9	3220	25·1
≥28.0	1192	9·3
Missing	141	1·1
Maternal ethnicity		
Han	12033	93·8
Other minorities	782	6·2
Educational level		
Primary/junior/high school education	5449	43·4
College or higher education	7223	55·5
Missing	143	1·1
Annual household income per capital (RMB)		
<10,000	2354	18·4
10,000~20,000	2143	16·7
20,000~30,000	1968	15·4
20,000~50,000	2581	20·1
> 50,000	3593	28·0
Missing	176	1·4
Household registration		
Urban citizens	6974	54·1
Rural citizens	5883	45·9
Insurance type		
Social and medical insurance	11908	92·9
Commercial medical insurance	135	1·1
None insurance	606	4·7

NA	166	1·3
**Geographic location**		
Southeast	4127	32·2
Southwest	1922	15·0
Central	5974	46·6
Northeast	792	6·2
**HDI**		
Very high	3357	26·2
High	2667	20·8
Upper middle	1677	13·1
Low middle	3192	24·9
Low	515	4·0
Very low	1407	11·0

Table S4 Prevalence estimates of gynecological diseases and STDs by HDI in women aged 20-49 years in 2020 CFSW: Overall population

	Overall<br>% (95%CI)	Very low<br>% (95%CI)	Low<br>% (95%CI)	Lower middle<br>% (95%CI)	Upper middle<br>% (95%CI)	High<br>% (95%CI)	Very high<br>% (95%CI)
**Gynecological diseases**							
Overall	49·41 (47·74-51·21)	47·65 (43·88-51·72)	39·30 (34·12-45·16)	44·50 (42·10-47·07)	54·48 (50·30-59·22)	56·11 (53·11-59·36)	44·98 (42·66-47·50)
Vulvovaginitis	31·63 (30·30-33·10)	26·63 (23·87-29·67)	25·60 (21·45-30·44)	25·18 (23·39-27·13)	33·83 (30·57-37·65)	41·18 (38·62-44·00)	28·05 (26·17-30·14)
Cervicitis	20·62 (19·58-21·80)	21·75 (19·18-24·62)	13·40 (10·46-17·04)	16·90 (15·45-18·50)	22·70 (20·04-25·93)	27·04 (25·11-29·22)	15·45 (14·17-16·94)
Pelvic infection diseases	13·17 (12·35-14·11)	14·45 (12·41-16·79)	11·31 (8·64-14·68)	13·11 (11·81-14·57)	12·14 (10·32-14·55)	15·73 (14·24-17·47)	10·28 (9·20-11·59)
Uterine myoma	8·23 (7·59-9·01)	5·27 (3·99-6·88)	3·67 (2·24-5·85)	5·34 (4·52-6·32)	10·77 (9·03-13·12)	11·76 (10·55-13·22)	8·44 (7·54-9·56)
Diminished ovarian reserve	2·70 (2·33-3·23)	5·92 (4·66-7·50)	1·14 (0·42-2·69)	2·97 (2·38-3·73)	3·05 (2·10-4·71)	2·35 (1·72-3·30)	1·54 (1·17-2·17)
Endometriosis	2·44 (2·11-2·92)	3·27 (2·32-4·56)	1·67 (0·76-3·37)	2·26 (1·76-2·92)	2·73 (1·98-4·20)	2·48 (1·88-3·38)	2·67 (2·14-3·44)
Genital malformation	0·29 (0·16-0·64)	0·41 (0·13-1·06)	0·19 (0·00-1·37)	0·08 (0·02-0·41)	0·31 (0·09-1·54)	0·53 (0·17-1·33)	0·11 (0·03-0·59)
Genital tuberculosis	0·22 (0·11-0·56)	0·32 (0·06-1·02)	0·19 (0·00-1·35)	0·08 (0·02-0·40)	0·17 (0·02-1·40)	0·43 (0·15-1·13)	0·07 (0·01-0·54)
**STD**							
Overall	2·23 (1·93-2·69)	1·56 (0·94-2·51)	0·37 (0·04-1·63)	1·54 (1·10-2·16)	1·52 (0·98-2·85)	5·33 (4·44-6·50)	0·60 (0·37-1·14)
Mycoplasma genitalium infection	1·67 (1·43-2·08)	1·21 (0·66-2·12)	0·19 (0·00-1·37)	1·02 (0·67-1·56)	0·80 (0·47-2·01)	4·60 (3·80-5·68)	0·25 (0·11-0·74)
Condyloma acuminatum	0·27 (0·15-0·61)	0·24 (0·04-0·86)	0·18 (0·00-1·34)	0·19 (0·05-0·58)	0·26 (0·06-1·49)	0·41 (0·13-1·13)	0·23 (0·10-0·72)
Syphilis	0·20 (0·10-0·54)	0·18 (0·02-0·79)	0·00 (NaN)	0·11 (0·03-0·44)	0·21 (0·04-1·44)	0·44 (0·15-1·15)	0·07 (0·01-0·55)
Chlamydia trachomatis genital infection	0·28 (0·16-0·62)	0·23 (0·04-0·84)	0·00 (NaN)	0·36 (0·14-0·82)	0·13 (0·03-1·33)	0·58 (0·21-1·38)	0·09 (0·02-0·57)
Toxoplasma gondii and cytomegalovirus	0·14 (0·07-0·47)	0·26 (0·05-0·89)	0·00 (NaN)	0·10 (0·02-0·43)	0·09 (0·01-1·30)	0·32 (0·07-1·02)	0·10 (0·03-0·57)
Genital herpes	0·14 (0·05-0·47)	0·13 (0·00-0·73)	0·00 (NaN)	0·08 (0·02-0·40)	0·16 (0·01-1·39)	0·29 (0·06-0·99)	0·05 (0·01-0·52)
Gonorrhea	0·13 (0·04-0·47)	0·13 (0·00-0·73)	0·00 (NaN)	0·05 (0·01-0·37)	0·05 (0·00-1·27)	0·40 (0·09-1·20)	0·02 (0·00-0·50)

Table S5 Prevalence estimates of gynecological diseases and STDs by HDI in women aged 20-49 years in 2020 CFSW: Population in Urban areas

	Overall %(95%CI)	Very low %(95%CI)	Low %(95%CI)	Lower middle %(95%CI)	Upper-middle %(95%CI)	High %(95%CI)	Very-high %(95%CI)
**Gynecological diseases**							
Overall	48·49 (45·91-51·44)	48·57 (43·87-54·19)	39·72 (31·02-51·64)	44·25 (40·66-48·43)	56·05 (49·78-63·91)	50·40 (45·98-55·42)	44·30 (41·39-47·57)
Vulvovaginitis	29·08 (27·00-31·55)	27·68 (24·23-32·08)	26·24 (19·02-36·87)	24·66 (22·07-27·86)	33·83 (28·8-40·54)	30·70 (27·19-34·86)	26·5 (24·19-29·18)
Cervicitis	18·47 (16·97-20·36)	21·49 (18·35-25·61)	9·26 (5·64-16·60)	16·72 (14·58-19·49)	24·31 (20·3-30·03)	19·31 (16·73-22·53)	14·56 (13·00-16·48)
Pelvic infection diseases	11·59 (10·47-13·12)	13·16 (10·8-16·54)	12·10 (7·89-19·89)	12·29 (10·49-14·74)	10·08 (7·96-14·11)	12·49 (10·44-15·20)	8·37 (7·10-10·04)
Uterine myoma	8·84 (7·81-10·29)	5·98 (4·49-8·61)	5·64 (2·90-12·38)	6·04 (4·80-8·00)	12·73 (10·08-17·24)	9·84 (7·97-12·41)	9·40 (8·15-11·05)
Diminished ovarian reserve	3·13 (2·58-4·19)	6·55 (4·81-9·44)	2·21 (0·72-8·31)	2·72 (1·80-4·46)	3·17 (2·05-6·62)	3·39 (2·33-5·23)	1·66 (1·19-2·63)
Endometriosis	3·21 (2·65-4·26)	4·55 (3·02-7·3)	2·71 (0·99-8·89)	2·69 (1·98-4·20)	3·40 (2·27-6·84)	3·43 (2·35-5·29)	3·12 (2·36-4·34)
Genital malformation	0·30 (0·10-1·22)	0·51 (0·16-2·53)	0·00 (NaN)	0·04 (0·00-1·40)	0·00 (NaN)	1·03 (0·26-2·90)	0·11 (0·01-0·95)
Genital tuberculosis	0·28 (0·10-1·19)	0·38 (0·07-2·43)	0·00 (NaN)	0·15 (0·03-1·49)	0·22 (0·01-3·68)	0·66 (0·13-2·29)	0·00 (NaN)
**STD**							
Overall	1·52 (1·13-2·47)	1·45 (0·80-3·54)	0·42 (0·01-6·32)	1·01 (0·59-2·37)	1·65 (0·89-5·01)	2·72 (1·62-4·68)	0·67 (0·37-1·54)
Mycoplasma genitalium infection	0·94 (0·65-1·84)	1·04 (0·48-3·1)	0·42 (0·01-6·32)	0·69 (0·35-2·04)	0·70 (0·30-4·03)	1·85 (1·01-3·57)	0·30 (0·12-1·14)
Condyloma acuminatum	0·36 (0·16-1·27)	0·35 (0·07-2·38)	0·00 (NaN)	0·15 (0·03-1·49)	0·51 (0·12-3·92)	0·66 (0·13-2·29)	0·19 (0·05-1·02)
Syphilis	0·24 (0·08-1·14)	0·15 (0·00-2·20)	0·00 (NaN)	0·10 (0·01-1·45)	0·09 (0·00-3·54)	0·72 (0·17-2·36)	0·07 (0·00-0·91)
Chlamydia trachomatis genital infection	0·28 (0·12-1·18)	0·30 (0·05-2·33)	0·00 (NaN)	0·12 (0·01-1·47)	0·13 (0·02-3·57)	0·79 (0·21-2·42)	0·10 (0·01-0·94)
Toxoplasma gondii and cytomegalovirus	0·21 (0·06-1·12)	0·22 (0·02-2·26)	0·00 (NaN)	0·06 (0·00-1·42)	0·17 (0·02-3·60)	0·53 (0·06-2·17)	0·08 (0·01-0·91)
Genital herpes	0·23 (0·07-1·15)	0·15 (0·00-2·20)	0·00 (NaN)	0·07 (0·00-1·43)	0·29 (0·02-3·73)	0·53 (0·06-2·17)	0·04 (0·00-0·87)
Gonorrhea	0·24 (0·06-1·17)	0·15 (0·00-2·20)	0·00 (NaN)	0·06 (0·00-1·42)	0·00 (NaN)	0·89 (0·18-2·77)	0·00 (NaN)

Table S6 Prevalence estimates of gynecological diseases and STDs by HDI in women aged 20-49 years in 2020 CFSW: Population in rural areas

	Overall %(95%CI)	Very low %(95%CI)	Low %(95%CI)	Lower middle %(95%CI)	Upper-middle %(95%CI)	High %(95%CI)	Very-high %(95%CI)
**Gynecological diseases**							
Overall	49·48 (46·86-51·74)	44·85 (36·25-55·58)	39·48 (32·85-47·13)	43·20 (39·77-46·91)	52·11 (46·49-58·76)	59·42 (55·24-64·06)	46·26 (42·34-50·7)
Vulvovaginitis	32·77 (30·95-34·82)	22·42 (16·75-30·26)	25·56 (20·32-31·82)	25·06 (22·49-27·89)	33·28 (29·02-38·57)	47·27 (43·64-51·37)	30·74(27·46-34·57)
Cervicitis	21·81 (20·29-23·57)	22·17 (16·03-30·61)	15·83 (11·78-20·92)	16·51 (14·42-18·87)	20·96 (17·34-25·71)	32·29 (29·47-35·57)	17·08(14·83-19·89)
Pelvic infection diseases	14·59 (13·36-16·07)	18·60 (12·94-26·61)	10·80 (7·48-15·17)	13·34 (11·46-15·5)	13·98 (11·18-17·92)	17·84 (15·69-20·48)	13·65(11·62-16·25)
Uterine myoma	7·01 (6·23-8·05)	1·38 (0·41-5·28)	2·33 (0·99-4·78)	4·42 (3·25-5·91)	8·64 (6·56-11·91)	12·12 (10·64-14·06)	7·08 (5·81-8·91)
Diminished ovarian reserve	2·34 (1·82-3·18)	5·60 (2·78-11·02)	0·25 (0·01-1·79)	3·28 (2·41-4·43)	2·82 (1·5-5·59)	1·50 (0·80-2·89)	1·42 (0·83-2·75)
Endometriosis	1·49 (1·14-2·16)	0·34 (0·06-3·99)	0·81 (0·17-2·64)	1·50 (0·94-2·35)	1·87 (1·02-4·21)	1·63 (1·05-2·85)	2·23 (1·49-3·65)
Genital malformation	0·29 (0·12-0·89)	0·09 (0·00-3·80)	0·37 (0·01-2·08)	0·13 (0·01-0·63)	0·62 (0·17-2·84)	0·12 (0·01-1·14)	0·08 (0·01-1·21)
Genital tuberculosis	0·13 (0·04-0·70)	0·00 (NaN)	0·31 (0·01-1·92)	0·00 (NaN)	0·10 (0·00-2·28)	0·21 (0·06-1·22)	0·16 (0·03-1·29)
**STD**							
Overall	2·68 (2·24-3·41)	2·11 (0·49-6·76)	0·25 (0·01-1·79)	1·93 (1·28-2·88)	1·30 (0·64-3·54)	7·01 (5·72-8·81)	0·41 (0·15-1·55)
Mycoplasma genitalium infection	2·16 (1·78-2·84)	1·69 (0·28-6·26)	0·00 (NaN)	1·20 (0·71-1·99)	0·87 (0·40-3·02)	6·36 (5·15-8·09)	0·13 (0·01-1·27)
Condyloma acuminatum	0·14 (0·05-0·71)	0·00 (NaN)	0·25 (0·01-1·79)	0·17 (0·02-0·72)	0·00 (NaN)	0·21 (0·03-1·27)	0·27 (0·08-1·4)
Syphilis	0·18 (0·06-0·76)	0·09 (0·00-3·80)	0·00 (NaN)	0·12 (0·01-0·61)	0·33 (0·03-2·57)	0·21 (0·03-1·27)	0·04 (0-1·17)
Chlamydia trachomatis genital infection	0·22 (0·09-0·81)	0·00 (NaN)	0·00 (NaN)	0·46 (0·16-1·09)	0·10 (0·00-2·28)	0·38 (0·04-1·64)	0·04 (0-1·17)
Toxoplasma gondii and cytomegalovirus	0·11 (0·04-0·68)	0·42 (0·01-4·25)	0·00 (NaN)	0·17 (0·02-0·72)	0·00 (NaN)	0·17 (0·02-1·21)	0·08 (0·01-1·21)
Genital herpes	0·04 (0·01-0·62)	0·00 (NaN)	0·00 (NaN)	0·12 (0·01-0·61)	0·00 (NaN)	0·06 (0·00-1·08)	0·04 (0-1·17)
Gonorrhea	0·04 (0·01-0·62)	0·00 (NaN)	0·00 (NaN)	0·06 (0·00-0·51)	0·10 (0·00-2·28)	0·00 (NaN)	0·04 (0-1·17)
